# Risk Factors for Visceral Leishmaniasis and Asymptomatic *Leishmania donovani* Infection in India and Nepal

**DOI:** 10.1371/journal.pone.0087641

**Published:** 2014-01-31

**Authors:** Albert Picado, Bart Ostyn, Shri Prakash Singh, Surendra Uranw, Epco Hasker, Suman Rijal, Shyam Sundar, Marleen Boelaert, François Chappuis

**Affiliations:** 1 Barcelona Centre for International Health Research (CRESIB, Hospital Clínic-Universitat de Barcelona), Barcelona, Spain; 2 Institute of Tropical Medicine, Antwerp, Belgium; 3 Banaras Hindu University, Varanasi, India; 4 BP Koirala Institute of Health Sciences, Dharan, Nepal; 5 Geneva University Hospitals, Geneva, Switzerland; Technion-Israel Institute of Technology Haifa 32000 Israel., Israel

## Abstract

There is increasing interest in the role of asymptomatic infection in transmission of Visceral Leishmaniasis (VL). We studied the individual, household and environmental factors associated with asymptomatic *Leishmania donovani* infected individuals and VL. 7,538 individuals living in VL endemic villages in India and Nepal were divided into three mutually exclusive groups based on their VL history and Direct Agglutination Test (DAT) results in yearly serosurveys over a two-year period. The groups were (1) VL cases, (2) asymptomatically infected individuals (seroconverters) and (3) seronegative individuals. VL cases and seroconverters were compared to seronegative individuals in mixed logistic regression models. The risk of seroconversion and disease was significantly increased in individuals aged 14 to 24 years old and by the presence of other DAT-positive, asymptomatically infected individuals and VL cases in the house. The risk of seroconversion was higher in Indian than in Nepalese villages and it increased significantly with age, but not so for VL. This study demonstrates that, when risk factors for leishmanial infection and VL disease are evaluated in the same population, epidemiological determinants for asymptomatic infection and VL are largely similar.

## Introduction

Visceral Leishmaniasis (VL), also known as kala azar, is one of the major public health concerns in large parts of north-east India, south-eastern Nepal and western Bangladesh. In the Indian subcontinent VL is caused by *Leishmania donovani*, a protozoan parasite transmitted by *Phlebotomus argentipes* in an anthroponotic cycle. According to official figures, in 2010 over 28,000 new cases were reported in these three countries [1]. However, this figure is an underestimation of the real number of cases [2] and falls far from the objective set by the regional governments to eliminate VL from the Indian subcontinent by 2015. VL tends to affect the poorest and marginalized communities [3] which may have difficulties accessing treatment [4]. VL clinical signs are characterized by fever, splenomegaly and pancytopenia and the disease tends to be fatal if it is not treated. Only part of the infected individuals eventually develop the disease [5]. However, asymptomatic *L. donovani* infected individuals may play an important role in sustaining transmission in endemic communities as suggested by mathematical modelling [6]. Currently VL control in the Indian subcontinent is based on detection and treatment of cases and vector control using indoor residual spraying. The poor implementation of these control measures [7], [8] may explain their limited impact. Knowing the factors associated with *L. donovani* infection and progression to disease could help designing more efficient control strategies.

There are a number of studies on risk factors for *L. donovani* infection and VL in the Indian subcontinent. In these studies VL patients or asymptomatically infected individuals are generally compared to healthy non-infected individuals respectively. Briefly, *L. donovani* infection, defined as positivity in serology or leishmanin skin test (LST) in a cross-sectional survey, has been associated with age, gender, house structure and socio-economic status as well as previous VL cases in the house or in the proximity [9]–[14]. There are fewer studies using seroconversion to define *L. donovani* infection [15], [16]. In India, the risk of seroconversion was associated with proximity to water bodies, humidity in the house, livestock ownership and religion. Some sleeping habits (e.g. use of a bednet, sleeping under a cover and inside) were found protective [15]. In other studies, the risk of clinical VL has been associated with individual (e.g. age, sleeping habits), household (e.g. mud house, other VL cases, DDT spraying), social (e.g. socio-economic status, caste) and environmental (e.g. cattle density) factors [10], [17]. According to these studies the risk factors for *L. donovani* infection and VL are not always concordant [10]. However it is difficult to conclude that they are completely different as the studies conducted so far used different methodology (i.e. different markers for infection, VL case definitions or study designs) and surveyed different populations, usually limited to single or few communities from India, Nepal or Bangladesh with relatively high and recent transmission. To properly compare the risk factors for *L. donovani* infection and clinical VL analysing them on the same population and over the same time frame is required. The aim of this study is to evaluate if individual, household and environmental factors associated with asymptomatic *L. donovani* infected individuals and VL clinical cases in India and Nepal are different. These two groups will be compared with the same population of controls, i.e. all the sero-negative individuals living in the same villages during the same timeframe.

## Materials and Methods

### Study Areas and Study Population

The subjects included in this study were selected from a large cohort of individuals followed for 2.5 years (from November 2006 to May 2009) as part of a cluster randomised controlled trial to evaluate the effect of longlasting insecticidal nets (LN) to prevent *L. donovani* infection and clinical VL in India and Nepal [18]. The clusters in the bednet trial were selected in 2006 based on their VL average annual incidence from 2003 to 2005 (minimum 0.8%). A cluster was either a hamlet or a ward with a population ranging from 350 to 1500 people. Further details on the trial methodology are provided elsewhere [18], [19]. The inhabitants from 20 clusters (14 in India and 6 in Nepal) where at least one VL case was identified from November 2006 to May 2009 were included in this risk factor study.

### *Leishmania donovani* Infections and VL Cases

*Leishmania donovani* infection was determined by direct agglutination test (DAT). Blood samples were collected by finger prick on filter papers (Whatman #3) from all individuals above 2 years old in three serosurveys conducted at a 12-months interval: November-December 2006, 2007 and 2008. Filter papers were eluted and the DAT was performed as described elsewhere [14]. The cut-off for DAT positivity was set at a titre of 1∶1600. Incident *L. donovani* infections were measured by seroconversion in the DAT in individuals who were DAT negative (≤1∶800) in their first blood sample. In addition, seroconversion was considered only if the difference between two DAT results was at least 2 titres to take into account the inter-reader variability of the DAT.

Incident VL cases were recorded during 3-monthly house-to-house surveys from November 2006 to May 2009. Individuals with fever for two weeks or more at the time of survey were examined by a physician and tested with Kalazar Detect Rapid Test (InBios International, Seattle, USA), a rapid serological test that proved to be highly sensitive and specific in South Asia [20]. Suspected VL cases were referred to the Kala-azar Medical Research Centre (KAMRC) in India and the B. P. Koirala Institute of Health Science (BPKIHS) in Nepal for further free VL diagnosis and treatment if required. VL cases who were treated between surveys were also included in the study; the data of these patients were collected retrospectively. Information on VL cases was collected using a structured questionnaire. For every death that was reported in between the surveys, a verbal autopsy was done by a physician to identify possible relation with VL. All VL cases identified were classified as possible, probable or certain incident cases by a clinician following the criteria listed in table 1.

**Table 1:** Visceral Leishmaniasis (VL) case definitions.

Visceral Leishmaniasis (VL) case category	Criteria (one or more per category)
*Certain VL*	A. Clinical suspect patient^1^ with positive bone marrow or spleen aspirate at one of the study reference hospitals^2^
	B. Clinical suspect patient^1^ with
	1. A positive rK39 dipstick^3^ (performed by the study team) AND
	2. Good clinical and haematological response to anti-leishmanial treatment given at study reference hospitals^2^
	C. Clinical suspect patient^1^ with
	1. A positive rK39 dipstick^3^ (performed by the study team) AND
	2. Good clinical response to anti-leishmanial treatment given outside study reference hospitals^2^ (e.g. other hospital, private physician) AND
	3. Retrospective confirmation of treatment from the care-provider^4^
	D. Clinical suspect patient^1^ with
	1. VL diagnosis (rK39 dipstick^3^, parasitology^5^) not performed by the study team AND
	2. Good clinical response to anti-leishmanial treatment given outside the study reference hospitals^2^ AND
	3. Retrospective confirmation of diagnosis and treatment from the care-provider^4^
	E. Patient who died of parasitologicaly proven^5^ visceral leishmaniasis.
*Probable VL*	A. Clinical suspect patient^1^ with
	1. A positive rK39 dipstick^3^ (performed by the study team) AND
	2. Good clinical response to anti-leishmanial treatment given outside the study reference hospitals^2^ AND
	3. No retrospective confirmation of treatment from the care-provider^4^
	B. Clinical suspect patient^1^ with
	1. Diagnosis (rK39 dipstick^3^, parasitology^5^) not performed by the study team AND
	2. Good clinical response to anti-leishmanial treatment given outside the study reference hospitals^2^ AND
	3. No retrospective confirmation of diagnosis and treatment from the care-provider^4^ AND
	4. Direct Agglutination Test (DAT) seroconversion^6^ at the following serological survey.
	C. Patient who died of probable visceral leishmaniasis.
*Possible VL*	A. Clinical suspect patients^1^ who are neither Certain nor Probable VL

1 Individual with history of fever for ≥ 2 weeks and splenomegaly.

2 Kala-Azar Medical Research Centre (KAMRC), Muzaffarpur in India and B.P. Koirala Institute of Health Sciences, Dharan in Nepal.

3 Kalazar Detect Rapid Test^TM^ (InBios International, Seattle, USA)

4 Verification of patient’s medical records.

5 Positive bone marrow or spleen aspirate

6 VL patients who seroconverted from DAT negative (≤1∶800) in the serosurvey before developing VL to DAT positive (>1∶800), with at least 2 titres difference, after developing the disease.

### Definitions

For the risk factor analyses the study population was divided into three exclusive groups following the definitions below. Individuals residing for less than 6 months/year in the study clusters were excluded from the analysis.

#### Visceral leishmaniasis cases

patients who suffered probable or certain primary VL (table 1) during the study period (November 2006 to May 2009). Relapse cases and PKDL patients were excluded. Similarly, VL cases infected before the study started were not included in this group. Thus VL cases who were DAT positive (>1∶800) in / or had VL symptoms starting prior to the first serosurvey (in November-December 2006) were also excluded.

#### Asymptomatic (subclinical) *L. donovani* infections

incident *L. donovani* infected individuals (that is seroconverters as defined above) from November 2006 to December 2008. Seroconverters who had previously suffered from VL were excluded. Seroconverters were followed for a minimum of 6 months; those developing clinical VL were excluded from this group and moved to the VL group (see above).

#### Non-infected individuals

individuals with three consecutive DAT-negative results, i.e. subjects who had DAT<1∶1600 in November-December 2006, 2007 and 2008. Individuals who did not have all 3 blood samples taken or had at least one positive result (i.e. DAT>1∶800) were excluded.

### Risk Factors

Information on potential risk factors at individual, household and neighbouring levels for the 3 study groups were collected in several house-to-house surveys from July 2006 to May 2009. The variables representing those risk factors are detailed below.

#### Individual factors

we recorded gender and age of all participants in July 2006. Age was categorized as follows: 0–6, 7–13, 14–24, 25–39 and 40+ (in years). In December-November 2006, during the first serosurvey, the height (in cm) and weight (in kg) were recorded. The nutritional status was determined based on the body mass index (BMI) for adults (>19 years old), BMI-for-age z-scores for individuals between 6 and 19 years old and weight for height z-scores for children 0 to 5 years old. Individuals were classified as normal, moderately or severely malnourished [14]. For analytical purposes the variable was dichotomised as normal or malnourished (moderately and severely malnourished were grouped together). The individual use of bednets (LN in intervention and non-treated nets in control clusters respectively) while sleeping at night was recorded at quarterly house- to-house surveys. Based on the data collected the frequency of use of bednets per individual was dichotomised as use over and below 80% of the nights.

#### Household factors

information on the type of house (i.e. thatched, mud, brick) was collected. For analytical purposes the variable was dichotomised: thatched vs. the rest of houses. A composite index was calculated to determine the socio-economic status (SES) of each household based on principal component analysis (PCA). This index included data on ownership of consumer durables, the characteristics of the house and of the people living in it. The index was divided into 5 quintiles to rank the households from the poorest to the richest [3], . Information on the indoor residual spraying (IRS) of the houses as part of the vector control programs of the ministries of health 18 months before and during the trial were recorded. For analytical purposes, houses were dichotomised as sprayed or not before and during the trial.

#### Presence of other VL, DAT positive and seroconverters in the house

In each house, the number of VL cases 18 months before and during the trial, the number of DAT-positive individuals at baseline and the number of seroconverters during the trial were recorded. The risk associated with the presence of other VL cases, DAT-positive individuals and seroconverters in the house was evaluated.

#### Environmental factors

To assess the effect of the presence of VL cases (before and during the trial), seroconverters and DAT-positives in the proximity, the number of these events 50 meters around each house was estimated. For analytical purposes the number of VL, DAT-positive individuals and seroconverters in the proximity were dichotomised as present or absent. The effect of population density as well as the density of domestic animal around the house was also estimated. Edge corrected kernel densities were calculated using the household data collected on the number of people, bovines (cows and buffaloes combined) and goats per house. A bandwidth of 50 m was used and the densities of the human population and that of domestic animals associated with each house were calculated using the package Spatstat in R 2.14.0 [17].

#### Other Factors

country (India or Nepal) and living in a cluster where LN were distributed as part of the bednet trial (intervention cluster) or in a control cluster (where no LN were distributed) were other factors considered in the analyses.

### Statistical Analyses

A mixed logistic regression model with cluster as random effect was used to compare the risk factors associated to *L. donovani* infection (DAT seroconversion) and VL. Firstly, risk factors associated with *L. donovani* infection and VL cases were determined by comparing asymptomatically infected individuals (seroconverters) and incident VL cases to non-infected individuals separately. Secondly, based on the previous results, the same regression model was applied to seroconverters and VL cases. This final model allowed evaluating if risk factors associated to asymptomatic *L. donovani* infected individuals and VL clinical cases were different.

A two-step procedure was used in the logistic regression models to determine the most parsimonious multivariate models. So the simplest models that best estimate the response variables. First the association of all covariates and the outcome was assessed one by one in bivariate analyses with cluster as random effect. In a second step, covariates with p-value ≤ 0.2 in the bivariate model were included in a multivariate mixed model. A backward stepwise selection method was applied to determine the most parsimonious models for seroconverters and VL cases separately. P-value ≤ 0.05 was used as threshold for removal of predictors from the model. Finally, the same logistic regression model including all factors identified in the most parsimonious models was applied to seroconverters and VL. Stata 12 was used in the analyses.

### Ethical Considerations

Written informed consent was obtained from head of households and individuals or their guardian for those aged under 18. The study protocol was revised and approved by the ethical committees of the Institute of Medical Sciences in India, B. P. Koirala Institute of Health Sciences in Nepal, the London School of Hygiene in the UK and Tropical Medicine and the University of Antwerp in Belgium.

## Results

**Figure 1:**
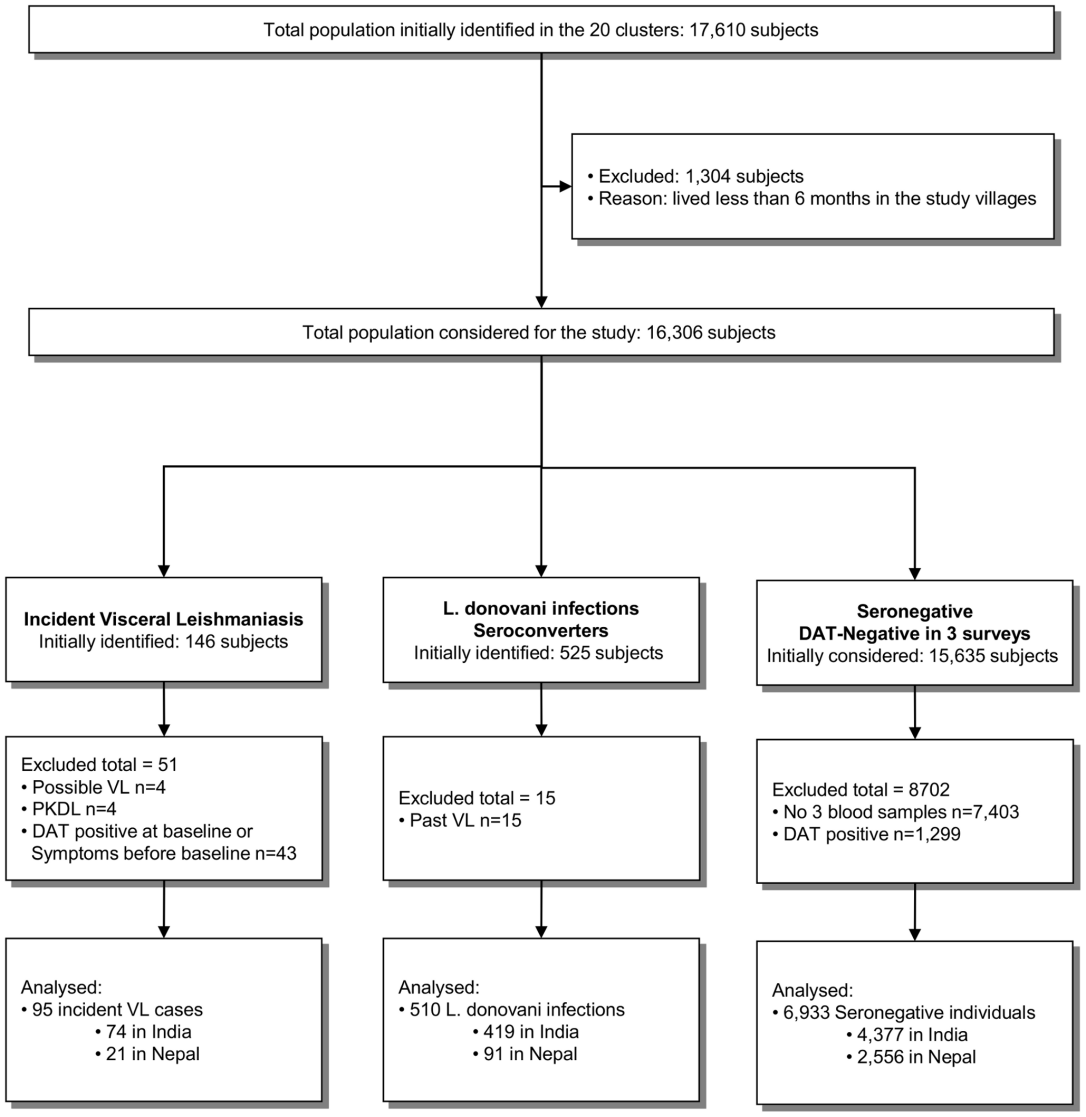
Population flow chart to allocate the subjects into the three study groups: (1) visceral leishmaniasis (VL) cases, (2) Seroconverters (asymptomatic *Leishmania donovani* infection) and (3) Seronegatives (DAT negative individuals).

Out of 17,610 individuals initially identified in the 20 clusters, 7,538 were finally included in this series of risk factors studies as detailed in the participants flow chart (Figure 1). 1,304 subjects were excluded because they lived less than 6 months in the study villages. In the VL group 51 subjects were excluded, mainly due to DAT-positive results at baseline or VL symptoms before this first survey (n = 43). In the seroconverters group 15 individuals were excluded as they were past VL cases. In the seronegative group 8,702 subjects had to be excluded from the analyses, mostly because they had less than 3 DAT results (n = 7,403). The 7,538 participants finally included in the analyses were divided as follows: 6,933 were considered non-infected individuals, 510 asymptomatically infected subjects (seroconverters) and 95 incident VL cases. The characteristics of the individuals allocated to the three study groups are detailed in Table 2.

**Table 2:** Characteristics of the individuals included in the risk factor studies divided by group: Seronegatives, Seroconverters (asymptomatic *Leishmania donovani* infection) and visceral leishmaniasis (VL) cases.

		Seronegatives	Seroconverters	VLcases
Total number of subjects		**6933**	**510**	**95**
India (%)		4377 (63.1)	419 (82.7)	74 (77.9)
Intervention clusters (%)		3820 (55.1)	293 (57.4)	44 (46.3)
*Individual Variables*				
Age (%)	0–6	1397 (20.1)	86 (16.9)	21 (22.1)
	7–13	1750 (25.2)	97 (19.0)	22 (23.2)
	14–24	879 (12.7)	83 (16.3)	19 (20.0)
	25–39	1265 (18.2)	98 (19.2)	15 (15.8)
	+40	1642 (23.7)	146 (28.6)	18 (18.9)
Males (%)		2870 (41.4)	210 (41.2)	56 (58.9)
Malnutrition (%)	Normal	5834 (84.1)	397 (77.8)	60 (63.2)
	Moderate-Severe	1051 (15.2)	83 (16.3)	7 (7.4)
	Missing	48 (0.7)	30 (5.9)	28 (29.5)
Individuals using nets >80% of nights (%)		4541 (65.5)	311 (61.0)	54 (56.8)
*Household Variables*				
Individuals living in thatched houses (%)		2358 (34.0)	227 (44.5)	44 (46.3)
Socio Economic Status (%)	1 (poorest)	1387 (20.0)	103 (20.2)	19 (20.0)
	2	1359 (19.6)	130 (25.5)	22 (23.2)
	3	1383 (19.9)	102 (20.0)	22 (23.2)
	4	1390 (20.0)	100 (19.6)	16 (16.8)
	5 (least poor)	1414 (20.4)	75 (14.7)	16 (16.8)
Individuals living in household sprayed ≤ 18 m before Nov 2006 (%)		2701 (39.0)	169 (33.1)	35 (36.8)
Individuals living in household sprayed from Nov 2006 – May 2009 (%)		3084 (44.5)	296 (58.0)	61 (64.2)
*Other VL. DAT+. Seroconverters in the house*				
Individuals living in houses with other VL cases 18 m before Nov 2006 (%)		556 (8.0)	70 (13.7)	14 (14.7)
Individuals living in houses with other DAT-positive individuals in Nov 2006 (%)		2336 (33.7)	250 (49.0)	59 (62.1)
Individuals living in houses with other VL cases between Nov 2006 – May 2009 (%)		276 (4.0)	51 (10.0)	20 (21.0)
Individuals living in houses with other Seroconverters between Nov 2006 – Nov 2008 (%)		1020 (14.7)	182 (35.7)	36 (37.9)
*Neighbouring factors*				
Other VL cases ≤ 18 m before Nov 2006 50 m around the house (%)		3750 (54.1)	310 (60.8)	70 (73.7)
Other DAT-positive individuals in Nov 2006 50 m around the house (%)		5965 (86.0)	474 (92.9)	88 (92.6)
Other VL cases between Nov 2006 – May 2009 50 m around the house (%)		3413 (49.2)	287 (56.3)	61 (64.2)
Other Seroconverters between Nov 2006 – Nov 2008 50 m around the house (%)		5240 (75.6)	450 (88.2)	91 (95.8)
Median density in m^2^ (Interquartile range)				
	People	5.14 (4.74; 5.6)	5.28 (4.79; 6.0)	5.41 (4.83; 5.7)
	Bovine	0.63 (0.35; 1.1)	0.66 (0.36; 1.0)	0.62 (0.44; 1.0)
	Goats	1.05 (0.65; 1.6)	0.97 (0.66; 1.6)	0.99 (0.67; 1.5)

The results of the bivariate (unadjusted) analyses and the most parsimonious multivariate mixed logistic regression models on the risk factors associated to seroconversion and VL are detailed in Tables S1 and S2 respectively. Briefly, the multivariate models showed that both the risk of seroconversion and VL were associated with age, socio-economic status, household spraying before the study and the presence of DAT-positive individuals, VL cases and seroconverters in the house. Additionally, seroconversion was associated with the country (significantly higher in India compared to Nepal) and VL was associated with gender and presence of seroconverters in the proximity of the house. All those factors were included in the final model applied to both seroconverters and VL cases (Table 3). The risk of seroconversion was higher in India (OR = 0.32 in Nepal), among individuals over 14 years of age (i.e. 14–24: OR = 2.19, 25–39: OR = 1.57 and over 40 years old: OR = 1.94) and for those living in households with low socio-economic status (OR = 0.63 in households in the highest SES quintile). The presence of other DAT-positive individuals (OR = 1.37), seroconverters (OR = 2.22) and VL cases (OR = 1.66) in the house and the spraying of the house 18 months before the baseline (OR = 1.53) also increased the risk of asymptomatic *L. donovani* infection. Similarly, the risk of VL was increased in males (OR = 0.42 in females) aged 14–24 years old (OR = 2.22) living in houses where other DAT-positive individuals (OR = 2.43), seroconverters (OR = 2.22) and VL cases (OR = 3.03) were present during the study period. Finally the presence of seroconverters in the proximity of the house was strongly associated with VL occurrence (OR = 8.73). The standard deviations of the random effects were 0.40 (P-value = 0.024) and 0.45 (P-value < 0.001) for the models evaluating risk factors for VL and seroconverters respectively. The inclusion of non-statistically significant variables to the most parsimonious models did not substantially modify the ORs observed (Table 3 and Table S2). Fitting a mixed logistic regression model with fixed effects for cluster (nested within country), and random effects for household to take into account the clustering of the data at this lower level did not significantly modify the results (data not shown).

**Table 3:** Risk factors for incident asymptomatically *L. donovani* infection (measured by seroconverters) and Visceral Leishmaniasis (VL) in VL endemic villages in India and Nepal compared to subjects who stayed DAT negative over 24 months.

		Seroconverters vs Seronegatives			VL cases vs Seronegatives		
Factors		OddsRatio	95% CI	P-value	OddsRatio	95% CI	P-value
Country							
	India	ref			ref		
	Nepal	**0.32**	**0.18; 0.58**	**<0.001**	0.92	0.43; 1.97	0.825
Gender							
	Male				ref		
	Female	0.89	0.73; 1.08	0.240	**0.42**	**0.27; 0.65**	**<0.001**
Age							
	0–6	ref			ref		
	07–13	1.00	0.74; 1.36	0.999	0.93	0.50; 1.73	0.822
	14–24	**2.19**	**1.57; 3.05**	**<0.001**	**2.22**	**1.14; 4.31**	**0.019**
	25–39	**1.57**	**1.14; 2.16**	**0.005**	1.40	0.69; 2.81	0.351
	over 40	**1.94**	**1.45; 2.59**	**0.000**	1.09	0.57; 2.09	0.800
Socio Economic Status							
	1 (poorest)	ref					
	2	0.98	0.74; 1.32	0.915	0.89	0.46; 1.72	0.725
	3	0.83	0.61; 1.13	0.232	0.93	0.48; 1.80	0.832
	4	0.83	0.61; 1.14	0.252	0.72	0.35; 1.47	0.363
	5 (least poor)	**0.63**	**0.45; 0.88**	**0.007**	0.81	0.40; 1.67	0.576
Household sprayed ≤ 18 m before Nov 2006		**1.53**	**1.06; 2.21**	**0.023**	1.49	0.80; 2.78	0.213
Presence of other DAT-positive individuals in the house in Nov 2006		**1.37**	**1.12; 1.67**	**0.002**	**2.43**	**1.55; 3.79**	**<0.001**
Presence of other VL cases in the house from Nov 2006 to May 2009		**1.66**	**1.16; 2.36**	**0.005**	**3.03**	**1.68; 5.48**	**<0.001**
Presence of other seroconverters in the house from Nov2006 to Nov 2008		**2.22**	**1.79; 2.75**	**<0.001**	**2.22**	**1.38; 3.58**	**0.001**
Presence of other seroconverters around the house from Nov2006 to Nov 2008		1.20	0.86; 1.68	0.273	**8.73**	**2.59; 29.41**	**<0.001**

Results from the logistic regression models with cluster as random effect to take into account the clustering of the data. All risk factors associated to seroconversion or VL cases identified in the most parsimonious models (see Table S1) were included in the final regression model. Statistically significant (P-value <0.05) results highlighted in the table (bold).

## Discussion

The risk factors associated with asymptomatic infection and clinical disease in VL endemic communities in India and Nepal were similar in this prospective cohort with 2.5 years follow-up. The risk of seroconversion and disease was significantly increased in individuals aged 14 to 24 years old and by the presence of other DAT-positive, asymptomatically infected individuals and VL cases in the house. These results contrast with the conclusions of a recent review on risk factors for VL in the Indian subcontinent stating that the epidemiological determinants associated to VL and subclinical infection may be different [10]. Our results also confirm that seroconversion and VL are strongly associated. There were however some differences. The risk of seroconversion was higher in Indian than Nepalese villages. A better socio-economic status was associated with a decreased risk of seroconversion. VL showed a similar trend but the results were not statistically significant; possibly due to lack of power (only 95 VL cases were included in the analysis). Finally, only VL was associated with the presence of *L. donovani* infected individuals around the house and only seroconversion was significantly associated with household spraying before the study period.

The results of this study should be compared with caution to previous risk factor studies as it has some particularities: (1) recent asymptomatic *L. donovani* infection was defined as incident DAT seroconversion whereas other studies would include prevalent antibody-positive persons; (2) seroconverters and VL cases were compared to subjects who stayed seronegative at three repeated surveys over a 2.5 year time interval. And (3) asymptomatic *L. donovani* infected individuals and VL clinical cases were both compared to the same concurrent comparison group (seronegative individuals) living in the same environment. This approach allowed us to evaluate if the epidemiological determinants associated with *L. donovani* infection and VL are different. However, selecting DAT-negative individuals as the “control” population required excluding a significant number of individuals with unknown serological status (Figure 1).

The risk of seroconversion was higher in India than Nepal but the risk of developing VL in infected persons was similar in both countries. This phenomenon, already described in a previous study [5], may be related to higher prevalence of VL cases in India compared to Nepal. The presence of VL cases in a household is strongly associated with *L. donovani* infection in other household members [10]. Other factors not considered in this study such as vector density or migration may also play a role. Individuals aged 14 to 24 years old had twice the risk of seroconverting and presenting with VL compared to children (0–6 years old). The risk associated with age has been described in previous studies both for *L. donovani* infection [12], [14] and VL [17]. In Bangladesh, getting older was associated with a decreased risk of suffering VL when clinical cases were compared with asymptomatic infections [10]. Similar results were obtained in this study as the risk of VL was around 1 (OR = 1.06) for individuals over 40 years compared with seronegative individuals. Men were found to be at higher risk of developing VL than women. Previous studies have reported this result for both types of VL, New World as well as Old World. The gender difference seems to be, at least partially, related to the role of sex hormones in modulating the anti-leishmania response [21].

Household characteristics have been used as proxy measures for sand fly exposure in humans. Poor housing (e.g. mud/thatched houses, cracked walls) and low socio-economic status have been identified as risk factors for *L. donovani* infection and VL [10], [17]. In the current study people living in thatched houses were not found to be at higher risk of seroconversion or VL than seronegative individuals. Belonging to the richer quintiles of the wealth distribution was associated with a reduced risk of infection. IRS in houses was associated with an increased risk of seroconversion. This association could be related to the fact that IRS, which is erratically applied and has no or limited effect [7], [19], is mainly conducted around previous VL cases which are, as shown in this study, clustered around asymptomatically infected individuals. Living in the same house or in the proximities of a VL case increased the risk of developing VL or being serology or LST positive [10]. This is the first study assessing the risk associated with clustering of asymptomatically infected individuals (defined by seroconversion) in the house and proximity. For both VL and asymptomatic infections, having another case or individual infected (seroconverter or DAT positive) in the house increased the risk. The presence of seroconverters in the proximity of the house (50 m) also increased the risk of developing VL when cases where compared with seronegative negative individuals.

Nevertheless results from risk factor analyses should be interpreted with caution. For example, the possible role of asymptomatic individuals in the transmission of *L. donovani* should be tested by experimental studies (i.e. xenodiagnosis). Risk factors associated with disease or infection may vary with time [22]and some factors were found to have contradictory effects on VL or *L. donovani* infection in previous studies. Two relevant examples are the role of domestic animals and the use of bednets. In some studies having domestic animals (mainly bovine) in or around the house was a risk factor [15], [23], whereas in others it was protective [13], [16], [24]. A matched case–control study specifically designed to assess the risk of VL if domestic animals were kept inside the house did not find any significant association [25]. Similarly, the use of bednets was found to be protective against VL and/or *L. donovani* infection in three studies [9], [15], [16] but did not show a significant effect in most other risk factor studies [11], [13], [17], [23], [26]. Several of these studies did not thoroughly control for confounding by socio-economic status, and it is a fact that bednet possession is much higher in the highest economic classes. LN did not significantly reduce the risk of *L. donovani* infection or VL in the only cluster randomised trial conducted to date [18]. Both factors, use of bednets and presence of domestic animals, were not associated with seroconversion or VL in the current study. Nutrition status, defined using WHO criteria, was not associated with VL occurrence in the current study. This finding contrasts with results from previous studies in Bangladesh [11] and Brazil [27] where nutritional factors (e.g. micronutrients) were associated with VL development. The large number of individuals for whom the nutritional status was not assessed in the VL group (29%) and the single anthropometric assessment (during the initial survey only) may have masked a possible association. A prospective study design, analysing individual nutritional and immunological indicators at baseline and at appropriate intervals, would be the only valid design to evaluate the risk of developing VL associated with those factors.

This study demonstrates that, when risk factors for leishmanial infection and VL disease are evaluated in the same population, epidemiological determinants for asymptomatic infection and VL are largely similar. Also, a strong association between the occurrence of a new VL case and the presence of asymptomatically infected persons in the proximity was established. Risk factor studies for leishmaniasis are complex because it is a disease with unstable transmission and because of the high number of latent infections for which validated markers are lacking. Still, future risk factor studies should preferably: (1) concentrate on incident cases of infection and disease, (2) carefully control for confounding by socio-economic status, sex and age and (3) take the clustered nature of data into account by proper statistical methods.

## Supporting Information

Table S1 **Bivariate (unadjusted) analyses; Risk factors for incident asymptomatically** ***L. donovani*** **infection (measured by seroconverters) and Visceral Leishmaniasis (VL) in VL endemic villages in India and Nepal compared to subjects who stayed DAT-negative over 24 months.** Results from the bivariate (unadjusted) logistic regression models with cluster as random effect.
(DOCX)

Table S2 **Most parsimonious models; Risk factors for incident asymptomatically** ***L. donovani*** **infection (measured by seroconverters) and Visceral Leishmaniasis (VL) in VL endemic villages in India and Nepal compared to subjects who stayed DAT-negative over 24 months.** Results from the logistic regression models with cluster as random effect.
(DOCX)
