# Supplementary material for: Risk Factors for Visceral Leishmaniasis and Asymptomatic Leishmania donovani Infection in India and Nepal
Source: PLoS One. 2014 Jan 31;9(1):e87641. doi: 10.1371/journal.pone.0087641 (PMC3909193; doi:10.1371/journal.pone.0087641)
Supplement: Table S1 — Bivariate (unadjusted) analyses; Risk factors for incident asymptomatically L. donovani infection (measured by seroconverters) and Visceral Leishmaniasis (VL) in VL endemic villages in India and Nepal compared to subjects who stayed DAT-negative over 24 months. Results from the bivariate (unadjusted) logistic regression models with cluster as random effect. (DOCX) [file pone.0087641.s001.docx]

|  |  | **Seroconverters vs Seronegatives** | | |  | **VL cases vs Seronegatives** | | |
| --- | --- | --- | --- | --- | --- | --- | --- | --- |
| **Factors** |  | **Odds**  **Ratio** | **95% CI** | **P-value** |  | **Odds**  **Ratio** | **95% CI** | **P-value** |
| Country |  |  |  |  |  |  |  |  |
|  | India | ref |  |  |  | ref |  |  |
|  | Nepal | 0.33 | (0.18; 0.59) | <0.001 |  | 0.48 | (0.24; 0.94) | 0.033 |
| Type of cluster during KALANET trial |  |  |  |  |  |  |  |  |
|  | Control | ref |  |  |  | ref |  |  |
|  | Intervention | 0.99 | (0.50; 1.99) | 0.999 |  | 0.66 | (0.35; 1.27) | 0.219 |
| Gender |  |  |  |  |  |  |  |  |
|  | Male | ref |  |  |  | ref |  |  |
|  | Female | 0.98 | (0.81; 1.18) | 0.845 |  | 0.48 | (0.32; 0.73) | 0.001 |
| Age |  |  |  |  |  |  |  |  |
|  | 0-6 | ref |  |  |  | ref |  |  |
|  | 07-13 | 0.96 | (0.71; 1.30) | 0.783 |  | 0.88 | (0.48; 1.62) | 0.693 |
|  | 14-24 | 1.92 | (1.39; 2.64) | <0.001 |  | 1.61 | (0.85; 3.05) | 0.140 |
|  | 25-39 | 1.40 | (1.03; 1.90) | 0.030 |  | 0.86 | (0.44; 1.69) | 0.673 |
|  | over 40 | 1.67 | (1.26; 2.21) | <0.001 |  | 0.78 | (0.41; 1.49) | 0.459 |
| Malnutrition |  |  |  |  |  |  |  |  |
|  | Normal | ref |  |  |  | ref |  |  |
|  | Mod./Severe | 1.05 | (0.82; 1.35) | 0.689 |  | 0.68 | (0.31; 1.50) | 0.340 |
| Using nets >80% of nights |  | 0.60 | (0.42; 0.84) | 0.003 |  | 0.69 | (0.41; 1.16) | 0.161 |
| Non-thatched house |  | 0.90 | (0.73; 1.10) | 0.299 |  | 0.65 | (0.42; 1.01) | 0.058 |
| Socio Economic Status |  |  |  |  |  |  |  |  |
|  | 1 (poorest) | ref |  |  |  | ref |  |  |
|  | 2 | 1.11 | (0.84; 1.47) | 0.459 |  | 1.04 | (0.55; 1.96) | 0.900 |
|  | 3 | 0.95 | (0.71; 1.290) | 0.765 |  | 0.99 | (0.53; 1.89) | 0.997 |
|  | 4 | 0.97 | (0.72; 1.32) | 0.862 |  | 0.79 | (0.40; 1.58) | 0.509 |
|  | 5 (least poor) | 0.77 | (0.55; 1.07) | 0.119 |  | 0.75 | (0.38; 1.51) | 0.424 |
| Household sprayed ≤ 18m before Nov 2006 |  | 1.37 | (0.91; 2.07) | 0.126 |  | 1.19 | (0.62; 2.25) | 0.600 |
| Household sprayed from Nov 2006 – May 2009 |  | 1.35 | (0.85; 2.14) | 0.198 |  | 2.13 | (1.24; 3.68) | 0.006 |
| Presence of other VL cases in the house ≤ 18m before Nov 2006 |  | 1.53 | (1.15; 2.02) | 0.003 |  | 1.54 | (0.84; 2.83) | 0.160 |
| Presence of other DAT-positive individuals in the house in Nov 2006 |  | 1.45 | (1.20; 1.76) | <0.001 |  | 3.05 | (1.98; 4.69) | <0.001 |
| Presence of other VL cases in the house from Nov 2006 to May 2009 |  | 2.64 | (1.90; 3.66) | <0.001 |  | 5.54 | (3.27; 9.38) | <0.001 |
| Presence of other seroconverters in the house from Nov 2006 to Nov 2008 |  | 2.34 | (1.91; 2.87) | <0.001 |  | 3.33 | (2.16; 5.15) | <0.001 |
| Presence of other VL cases around the house ≤ 18m before Nov 2006 |  | 0.95 | (0.74; 1.23) | 0.713 |  | 2.14 | (1.27; 3.62) | 0.004 |
| Presence of other DAT-positive individuals around the house in Nov 2006 |  | 1.07 | (0.70; 1.65) | 0.742 |  | 1.66 | (0.66; 4.15) | 0.280 |
| Presence of other VL cases around the house from Nov 2006 to May 2009 |  | 0.96 | (0.77; 1.19) | 0.719 |  | 1.56 | (0.97; 2.52) | 0.066 |
| Presence of other seroconverters around the house from Nov 2006 to Nov 2008 |  | 1.33 | (0.95; 1.84) | 0.092 |  | 9.25 | (2.84; 30.12) | <0.001 |
| People density (in m2) |  | 1.01 | (0.86; 1.19) | 0.868 |  | 1.18 | (0.88; 1.58) | 0.270 |
| Bovine density (in m2) |  | 0.88 | (0.73; 1.06) | 0.192 |  | 1.05 | (0.80; 1.38) | 0.722 |
| Goat density (in m2) |  | 1.01 | (0.91; 1.12) | 0.882 |  | 0.91 | (0.68; 1.22) | 0.535 |
