# Supplementary material for: Risk Factors for Visceral Leishmaniasis and Asymptomatic Leishmania donovani Infection in India and Nepal
Source: PLoS One. 2014 Jan 31;9(1):e87641. doi: 10.1371/journal.pone.0087641 (PMC3909193; doi:10.1371/journal.pone.0087641)
Supplement: Table S2 — Most parsimonious models; Risk factors for incident asymptomatically L. donovani infection (measured by seroconverters) and Visceral Leishmaniasis (VL) in VL endemic villages in India and Nepal compared to subjects who stayed DAT-negative over 24 months. Results from the logistic regression models with cluster as random effect. (DOCX) [file pone.0087641.s002.docx]

|  |  | **Seroconverters vs Seronegatives** | | |  | **VL cases vs Seronegatives** | | |
| --- | --- | --- | --- | --- | --- | --- | --- | --- |
| **Factors** |  | **Odds**  **Ratio** | **95% CI** | **P-value** |  | **Odds**  **Ratio** | **95% CI** | **P-value** |
| Country |  |  |  |  |  |  |  |  |
|  | India | ref |  |  |  |  |  |  |
|  | Nepal | 0.32 | (0.18; 0.55) | <0.001 |  | -- | -- | -- |
| Gender |  |  |  |  |  |  |  |  |
|  | Male |  |  |  |  | ref |  |  |
|  | Female | -- | -- | -- |  | 0.42 | (0.27; 0.64) | <0.001 |
| Age |  |  |  |  |  |  |  |  |
|  | 0-6 | ref |  |  |  | ref |  |  |
|  | 07-13 | 1.00 | (0.74; 1.36) | 0.976 |  | 0.93 | (0.50; 1.72) | 0.818 |
|  | 14-24 | 2.13 | (1.53; 2.95) | <0.001 |  | 2.15 | (1.12; 4.12) | 0.021 |
|  | 25-39 | 1.55 | (1.14; 2.11) | 0.006 |  | 1.40 | (0.69; 2.81) | 0.347 |
|  | over 40 | 1.92 | (1.44; 2.56) | <0.001 |  | 1.06 | (0.55; 2.03) | 0.858 |
| Socio Economic Status |  |  |  |  |  |  |  |  |
|  | 1 (poorest) | ref |  |  |  |  |  |  |
|  | 2 | 0.98 | (0.74; 1.31) | 0.916 |  | -- | -- | -- |
|  | 3 | 0.81 | (0.60; 1.10) | 0.184 |  | -- | -- | -- |
|  | 4 | 0.81 | (0.60; 1.10) | 0.182 |  | -- | -- | -- |
|  | 5 (least poor) | 0.61 | (0.44; 0.86) | 0.004 |  | -- | -- | -- |
| Household sprayed ≤ 18m before Nov 2006 |  | 1.54 | (1.07; 2.22) | 0.021 |  | -- | -- | -- |
| Presence of other DAT-positive individuals in the house in Nov 2006 |  | 1.39 | (1.15; 1.70) | 0.001 |  | 2.37 | (1.53; 3.67) | <0.001 |
| Presence of other VL cases in the house from Nov 2006 to May 2009 |  | 1.67 | (1.17; 2.37) | 0.004 |  | 3.09 | (1.71; 5.57) | <0.001 |
| Presence of other seroconverters in the house from Nov 2006 to Nov 2008 |  | 2.25 | (1.81; 2.79) | <0.001 |  | 2.20 | (1.38; 3.53) | 0.001 |
| Presence of other seroconverters around the house from Nov 2006 to Nov 2008 |  | -- | -- | -- |  | 8.47 | (2.01; 27.48) | <0.001 |
